# Mr. Hamilton on a Peculiarity in Vaccination

**Published:** 1812-12

**Authors:** W. Hamilton

**Affiliations:** Ipswich


*To the Editors of the Medical and Physical Journal.*

GENTLEMEN,

AS your correspondent, X. Y. Z., who transmitted you his remarkable case of Vaccination, wishes to be informed whether such an occurrence has been observed by any other practitioner; I beg leave to inform him, through the medium of your useful Journal, that two such instances have happened to me.

In one of these cases, finding no appearance of infection after a delay of five or six days, and presuming I had lost my matter, I sent to London, received in a few days a fresh supply, and was just about to apply the same, when I observed, to my surprise, a regular pustule, from which I inoculated several children with the usual success.

With respect to the cause of these remarkably slow appearances of the pock, were I to hazard an opinion, I should impute it to the dilute state of the matter when inserted, or some peculiarity of constitution; as most vaccinators must have observed a considerable variation in the period necessary for maturity, some being as far advanced in five days as others are at ten.

I remain, Gentlemen,
With great respect,
Your's, &c.

*Ipswich, Nov.* 3, 1812. W. HAMILTON.

